# Rethinking Bone Disease in Kidney Disease

**DOI:** 10.1002/jbm4.10117

**Published:** 2018-11-15

**Authors:** Matthew J Damasiewicz, Thomas L Nickolas

**Affiliations:** ^1^ Department of Nephrology Monash Health Clayton Australia; ^2^ Department of Medicine Monash University Clayton Australia; ^3^ Columbia University Medical Center Department of Medicine Division of Nephrology New York NY USA

**Keywords:** CKD, ESKD, CKD‐MBD, FRACTURES, OSTEOPOROSIS, BONE DISEASE

## Abstract

Renal osteodystrophy (ROD) is the bone component of chronic kidney disease mineral and bone disorder (CKD‐MBD). ROD affects bone quality and strength through the numerous hormonal and metabolic disturbances that occur in patients with kidney disease. Collectively these disorders in bone quality increase fracture risk in CKD patients compared with the general population. Fractures are a serious complication of kidney disease and are associated with higher morbidity and mortality compared with the general population. Furthermore, at a population level, fractures are at historically high levels in patients with end‐stage kidney disease (ESKD), whereas in contrast the general population has experienced a steady decline in fracture incidence rates. Based on these findings, it is clear that a paradigm shift is needed in our approach to diagnosing and managing ROD. In clinical practice, our ability to diagnose ROD and initiate antifracture treatments is impeded by the lack of accurate noninvasive methods that identify ROD type. The past decade has seen advances in the noninvasive measurement of bone quality and strength that have been studied in kidney disease patients. Below we review the current literature pertaining to the epidemiology, pathology, diagnosis, and management of ROD. We aim to highlight the pressing need for a greater awareness of this condition and the need for the implementation of strategies that prevent fractures in kidney disease patients. Research is needed for more accurate noninvasive assessment of ROD type, clinical studies of existing osteoporosis therapies in patients across the spectrum of kidney disease, and the development of CKD‐specific treatments. © 2018 The Authors. *JBMR Plus* published by Wiley Periodicals, Inc. on behalf of the American Society for Bone and Mineral Research.

## Introduction

Renal bone disease or renal osteodystrophy (ROD) is a complex disorder of bone in patients with chronic kidney disease (CKD).^1^, ^2^, ^3^ Progressive kidney disease results in metabolic and hormonal disturbances that impair bone quality and is characterized by abnormal remodeling (low, normal or high turnover) with or without abnormalities in mineralization. Altered bone and mineral metabolism in kidney disease is part of a broader systemic disorder defined by the Kidney Disease Improving Global Outcomes (KIDGO) as CKD‐mineral and bone disorder (CKD‐MBD).^4^ CKD‐MBD is manifested by either one or a combination of: 1) abnormalities of calcium, phosphate, parathyroid hormone (PTH), or vitamin D metabolism; 2) abnormalities of bone turnover, mineralization, volume or strength, and linear growth; and 3) vascular or soft tissue calcification. Manifestations of CKD‐MBD begin early in CKD, with near‐normal kidney function, and the severity of CKD‐MBD and its clinical outcomes of increased fracture risk increase in parallel with declining renal function.^5^, ^6^, ^7^

In practical terms, our ability to confidently diagnose ROD type in CKD and to initiate strategies that could prevent fractures remains limited by the lack of accurate and noninvasive diagnostic tools. The measurement of calcium, phosphate, vitamin D, and PTH identify metabolic abnormalities associated with CKD‐MBD, but these remain poor markers of ROD and have insufficient discrimination of ROD turnover type and mineralization.^8^, ^9^ Bone turnover markers (BTMs), routinely used in non‐CKD patients to monitor fracture risk and osteoporosis therapy, lack validation in CKD and end‐stage chronic kidney disease (ESKD) and are consequently used infrequently. Transiliac crest bone biopsy remains the gold standard tool to assess bone quality in metabolic bone diseases; however, bone biopsy is invasive, expensive, painful, requires time‐consuming measurements, and is available at only a few centers worldwide.

Over the past decade, advancements in the field of metabolic bone diseases have the potential to alter the paradigm of how we approach renal bone disease. In 2017, KDIGO updated its guidelines to recommend risk classification of patients with kidney disease for fracture by measurement of areal bone mineral density (BMD) by dual‐energy X‐ray absorptiometry (DXA).^10^ Noninvasive measurement of cortical and trabecular microarchitecture is now possible with both high‐resolution imaging techniques and novel algorithms that reinterpret grayscale variation in DXA images. The diagnostic utility of BTMs in kidney disease is also being refined and fracture risk assessment tools based on clinical risk factors alone have been developed. Below we review the state of the field pertaining to the epidemiology, pathology, diagnosis, and management of renal bone disease in patients with CKD 3‐5D. Furthermore, we provide our personal interpretation of the current issues and advocate for a greater clinical awareness of this condition and the pressing need to develop and test strategies to prevent fractures in these patients.

## Pathophysiology of Renal Osteodystrophy

ROD is the bone component of CKD‐MBD and is defined as alterations in bone morphology associated with progressive CKD that can be quantified by bone histomorphometry.^(4)^ It is important to note that although CKD is defined as an estimated glomerular filtration rate (eGFR) <60 mL/min/1.73m^2^, changes of CKD‐MBD are present even with mild renal impairment (eGFR 60–90 mL/min/1.73m^2^).^11^, ^12^, ^13^ The historically complex nomenclature pertaining to ROD was simplified by the KDIGO working group and aligned with the standard nomenclature recommended by the American Society for Bone and Mineral Research.^4^, ^14^ Classification of ROD type was based on the turnover (high, normal, or low), mineralization (normal or abnormal), and volume (high, normal, or low) of bone (TMV classification system, Table 1). The aim of the revised classification was to encompass the most significant bone abnormalities in kidney disease that would inform management decisions.

**Table 1:** Bone Turnover, Mineralization, and Volume (TMV) Classification System for Renal Osteodystrophy^a^

Turnover	Mineralization	Volume
Low		Low
	Normal	
Normal		Normal
	Abnormal	
High		High

Reprinted with permission from ^a^Moe et al.^4^

Historically, bone abnormalities in patients with kidney disease were attributed to alterations in PTH and 1,25 dihydroxyvitamin D [1,25(OH)_2_D]. The characterization of circulating and bone‐derived hormones and the changes that occur with progressive kidney disease have fundamentally altered our understanding of the changes of CKD‐MBD and the pathogenesis of ROD. A detailed discussion of these changes is beyond the scope of this review. However, progressive CKD is generally associated with increased levels of PTH, fibroblast growth factor 23 (FGF‐23), osteoprotegerin, sclerostin, and Dickkopf‐related protein 1 (DKK1) and reductions in α‐Klotho, serum 25‐hydroxyvitamin D [25(OH)D], and [1,25(OH)_2_D].^12^, ^13^, ^15^, ^16^, ^17^, ^18^, ^19^, ^20^, ^21^, ^22^ The expression of bone turnover such as bone‐specific alkaline phosphatase (BSAP), procollagen type 1 N‐terminal (P1NP), C‐terminal telopeptide of type 1 collagen (CTX), and tartrate‐resistant acid phosphatase (TRAP‐5b) is more variable and dependent not only on the degree of renal impairment but also bone turnover.^23^ Together, these changes impact bone formation, resorption, and mineralization through their effects on osteoblast and osteocyte function.

Expression of skeletal proteins such as FGF‐23, dentin matrix protein 1, and matrix extracellular phosphoglycoprotein is also altered in CKD. In a study by Periera and colleagues,^24^ FGF‐23 and DMP1 expression was increased across all stages of CKD (compared with healthy controls), whereas there was no difference in MEPE expression. FGF‐23 and DMP1 were inversely related to osteoid accumulation, whereas MEPE was inversely related to bone volume, suggesting a role for FGF‐23 and DMP1 in bone mineralization and MEPE in the regulation of bone volume. The Wnt/β‐catenin pathway is essential for normal osteoblast differentiation and function and therefore normal bone formation. Sclerostin and Dickkopf‐1 (DKK1) are two circulating inhibitors of this pathway; these inhibit lipoprotein receptor‐related protein 5/6 activation of Wnt signaling and impair normal osteoblast differentiation.^21^, ^25^, ^26^, ^27^ Sclerostin is primarily expressed in skeletal tissue, and its expression is maintained during aging; in contrast, expression of DKK1 is more general and decreases in bone with age.^28^, ^29^, ^30^ Inhibition of sclerostin and DKK1 leads to increased bone formation in humans and animal models.^31^, ^32^, ^33^, ^34^, ^35^ Furthermore, animal studies suggest that DKK1 overexpression negatively impacts bone healing, suggesting a role for DKK1 inhibition during the fracture repair process.^36^, ^37^ Sclerostin and DKK1 levels are elevated in CKD, with sclerostin levels increased early in CKD, and generally preceding the rise of FGF‐23 and β‐catenin.^27^, ^38^ In a study of ESKD patients, sclerostin levels were inversely associated with reduced bone formation and bone loss over a 1‐year period.^39^, ^40^ These studies highlight the complex endocrine and paracrine bone‐renal signaling pathways and suggest that the pathogenesis of ROD is driven by changes within osteocytes that occur early in CKD.

In 2001, the National Institutes of Health defined osteoporosis as a skeletal disorder characterized by compromised bone strength, predisposing to an increased risk of fracture.^41^ Bone strength is a combined measure that reflects both bone density and quality. Bone density can be determined by DXA; however, bone quality is more broadly defined and pertains to bone material properties, such as bone remodeling, microdamage, microarchitecture, and collagen and mineral characteristics.^42^ Disorders of bone quality accumulate with age and directly affect the mechanical properties of bone and therefore fracture risk. In health, damaged areas of bone are constantly targeted for ongoing remodeling and repair. In CKD, some or all aspects of bone quality may be impaired (Table 2).^43^, ^44^, ^45^, ^46^, ^47^, ^48^ This includes defective mineralization (osteomalacia), abnormal remodeling (low‐ or high‐turnover bone disease), increased microarchitectural impairment (cortical porosity), and accumulation of microdamage and advanced glycation end products (AGE) cross‐linking.^43^, ^44^

**Table 2:** Bone Changes Associated With Hormonal and Metabolic Changes of End‐Stage Kidney Disease

Decreased bone density
Alterations in bone microarchitecture
• Cortical porosity
• Cortical thinning and trabecularization
• Trabecular thinning and dropout
• Disruption in balance and orientation of newly formed and mature bone
Decreased bone quality
• Mineralization (osteomalacia)
• Abnormal remodeling (loss of normal repair processes)
о Adynamic bone disease
о Low turnover
о High turnover
• Microdamage accumulation
о Reduced resistance to impact
• Advanced glycation end products cross‐linking
о Loss of elasticity and tissue embrittlement

Kidney disease patients have both traditional and CKD‐specific risk factors for bone disease and fractures (Table 3). For example, older age, low body weight, postmenopausal status, a history of previous fractures, increased risk of falls, and the use of immunosuppressive medications that promote bone loss are all common in CKD cohorts.^49^, ^50^, ^51^, ^52^ Metabolic disturbances that occur due to CKD, including decreased levels of nutritional and activated vitamin D, disordered calcium and phosphorous metabolism, premature hypogonadism, hyperparathyroidism, and metabolic acidosis all contribute to abnormalities in bone strength.^4^, ^53^, ^54^, ^55^, ^56^ Patients with kidney disease are also more likely to have reduced physical activity, postural hypotension, and decreased muscle mass, which increase susceptibility to falls and fractures.^57^, ^58^ The age‐specific risk of fracture associated with CKD is higher in younger age groups, but the absolute risk of fracture increases with age, suggesting an interaction between CKD‐specific and traditional risk factors for fracture in older CKD and ESKD patients.^7^, ^59^, ^60^

**Table 3:** General and CKD‐Specific Risk Factors for Bone Loss and Fractures

General risk factors	CKD‐specific
Patient‐related (non‐modifiable)	• Hyperparathyrodism
• Age	• Low nutritional and activated vitamin D
• Sex	• Disordered mineral metabolism
• Ethnicity	• Chronic inflammation
• Past history of fracture	• Metabolic acidosis
	• Premature hypogonadism
General (modifiable)	• Medications
• Low physical activity	о Steroids
• Smoking	о Phosphate binders (eg, aluminium)
• Alcohol	о CNI
• Medications (eg, streoids)	• Dietary restriction
• Diabetes	• Dialysis‐related amyloidosis
• Sarcopenia	
• Chronic inflammatory disorders	• Higher prevalence of general risk factors for osteoporosis

CKD = chronic kidney disease; CNI = calcineurin inhibitor.

Bone biopsy studies in CKD patients have provided important insights into the patterns of ROD observed in patients across the spectrum of CKD. In patients with CKD stages 3 to 5 (non‐dialysis), some data suggest that up to three‐quarters of patients have histologic evidence of renal osteodystrophy.^1^, ^61^, ^62^, ^63^, ^64^, ^65^, ^66^ Depending on the cohort studied, there is considerable variation in the prevalence of ROD types, and findings include a predominance of high or adynamic bone disease and even normal bone. In ESKD, recent large bone biopsy studies have characterized tissue‐level impairments in bone quality that reflect current CKD‐MBD management strategies. In a seminal study, Malluche and colleagues used the TMV classification system to evaluate 630 bone biopsies from adult hemodialysis patients from Europe and the United States.^9^ For turnover, 58%, 25%, and 18% of patients had low, high, and normal turnover, respectively. There were clear racial differences in turnover: low bone turnover predominated in whites (62%) and normal or high turnover predominated in blacks (68%). For mineralization, defects were uncommon (3% of patients). For volume, low, normal, or high cancellous bone volume was equally distributed among whites, but high volume predominated in blacks. Furthermore, blacks had normal cortical thickness with higher porosity, but whites had an equal distribution of low or normal thickness with high or normal porosity. Trabecular microarchitecture was also examined, with trabecular thickness being low in 37% of patients, normal in 40% of patients, and high in 13% of patients. Trabecular separation was normal in most (78%) of patients. Interestingly, both black and white patients with high bone turnover had increased porosity, and more than 80% of patients with low cancellous bone volume had thin trabeculae. These findings were supported in a recent study, where bone histomorphometric assessment of turnover (bone formation rate/bone surface [BFR/BS]) was performed in 492 dialysis patients.^8^ Low turnover was the dominant lesion being present in 59% (*n* = 289), whereas high turnover was present in 17% (*n* = 83). In a smaller study of 35 hemodialysis patients, those with low bone turnover had more microstructural abnormalities (lower cancellous bone volume and trabecular thickness) than those with high or normal turnover.^67^ Conversely, those with high turnover had reduced mineral content and reduced stiffness.

These data suggest that early CKD is characterized by high bone turnover. In ESKD, low‐turnover disease is the dominant lesion; however, high‐turnover lesions are present in a significant proportion of patients, whereas mineralization defects are comparably low in both groups. Importantly, the studies above highlight that ROD is more than bone turnover but a global disorder of bone quality and strength. It has been proposed that ROD and fractures related to kidney disease be considered as a subtype of osteoporosis (analogous to steroid‐induced osteoporosis), as bone quality and strength are impaired to a greater extent than age‐related osteoporosis.^68^ Although intentionally provocative, this definition highlights the need for a more practical and easily translatable definition of ROD. This should not only incorporate bone biopsy findings but surrogate parameters of bone strength (such as BTMs, DXA) that are easily accessible in daily clinical practice, facilitate diagnosis and inclusion in therapeutic trials, and inform treatment decisions.

## Fracture Epidemiology in CKD and ESKD

The incidence and prevalence of fractures increases with CKD stage and has been reported to be 2‐ to 17‐fold higher in CKD patients compared with the general population.^7^, ^59^, ^69^, ^70^, ^71^, ^72^ Recent studies have provided important insights into secular trends of fracture epidemiology in ESKD, highlighting differences in fracture rates between general population and ESKD cohorts and in fracture incidence rates at the central and peripheral skeleton.

**Figure 1:**
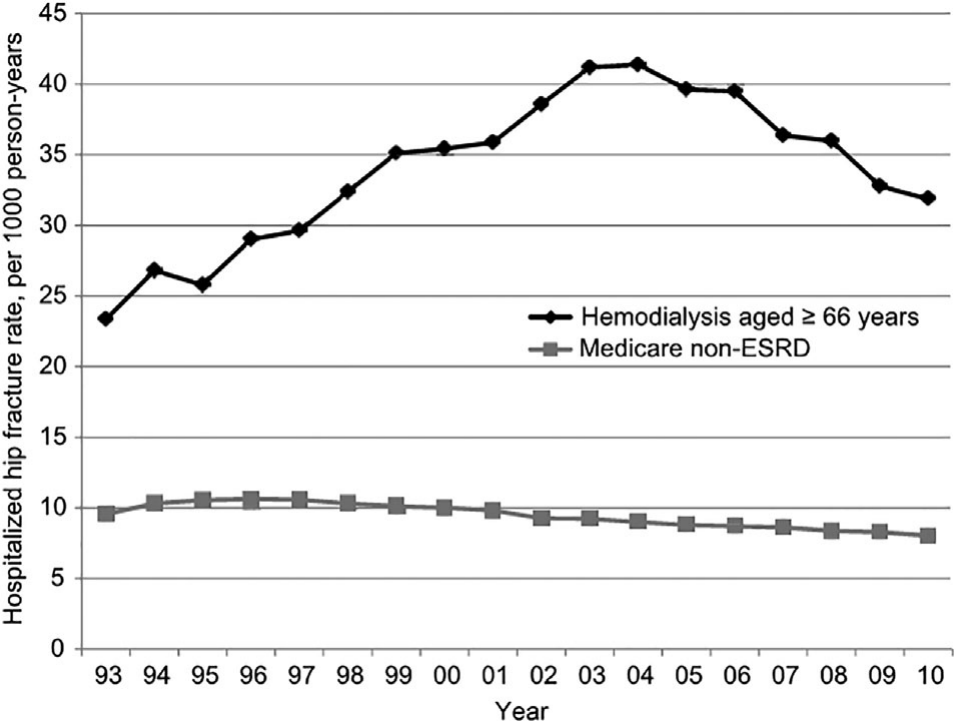
Adjusted hospitalized fracture rates per 1000 person‐years in Medicare point‐prevalent hemodialysis and non‐ESKD patients aged 66 years or older. Reprinted with permission from Arneson et al.^73^

Longitudinal studies using USRDS and US Medicare data have compared the incidence of hip fractures in ESKD and non‐ESKD cohorts.^73^, ^74^ In general, hip fracture rates in ESKD increased steadily from the early 1990s until the mid 2000s (an increase of 43%), with a nonsignificant reduction in incidence after 2004 (Fig. 1).^73^ More recent data from the US Nationwide Inpatient Sample examined age‐ and sex‐standardized hip fracture rates in ESKD and found a 12.6% decrease between 2003 and 2011.^75^ However, in 2010, hip fracture rates in ESKD remained 27% higher compared with 1996, and individuals aged 66 years or older with ESKD had a markedly higher incidence of hip fractures compared with those without ESKD (31.9 versus 8.0 per 1000 patient‐years). Furthermore, although these data suggest that hip fracture rates are decreasing, peripheral (arm and leg) fractures have more than doubled over the corresponding period.^60^ It is important to put these data in perspective, and the small decrease in hip fractures reported must be interpreted in the context of a persistently high overall fracture rate in ESKD patients compared with the general population. All studies report that current fracture rates are significantly higher in ESKD compared with general population cohorts, and fractures remain more prevalent today than they were in 1996.

The morbidity, mortality, and health care costs associated with fractures are higher for patients with kidney disease compared with the general population.^68^ Patients with CKD and ESKD experience longer hospitalization after a fracture, with reported mortality rates between 16% and 60%.^74^, ^76^, ^77^ Many patients do not return to their premorbid level of function after a hip fracture, with as many as 80% discharged to a long‐term‐care facility after a fracture.^5^ It was estimated that in 2010 hip fracture‐associated expenses in patients with CKD and ESKD were in excess of $600 million USD,^(5)^ and in the general US population, the costs of fractures and associated morbidity are projected to increase to more than $25 billion USD by 2025.^78^

## Noninvasive Assessment of Bone Quality and Fracture Risk in CKD

Bone biopsy is the gold standard for assessing bone quality in kidney disease and informs treatment options based on bone formation rates and mineralization characteristics. However, its utility as an everyday clinical tool is limited by lack of availability and long duration of time required to process and analyze bone tissue. Therefore, there is great interest in the use of noninvasive approaches to assess bone quality in kidney‐related bone disease so that fracture risk classification and the selection of antifracture treatments can be implemented broadly in the renal clinic. Imaging modalities such as DXA, Trabecular bone score (TBS), conventional Quantitative computed tomography (QCT), High‐resolution peripheral QCT (HR‐pQCT) and micro magnetic resonance imaging (MRI) assess bone density and/or structural aspects of bone quality, whereas PTH and BTMs assess aspects of bone quality that cannot be assessed by imaging, such as bone formation rates and mineralization. We will discuss the role of each of these in CKD.

## Bone Density and the Fracture Risk Assessment Tool

DXA is the clinical standard to determine BMD and measure fracture risk in the general population and is integral to the World Health Organization definition of osteoporosis (*T*‐score ≤–2.5).^79^ Historically the role of DXA to assess bone health and fracture risk in CKD3‐5D was controversial, as small cross‐sectional studies did not demonstrate that DXA discriminated prevalent fractures, and BMD measurements did not predict type of ROD. However, several recent longitudinal studies in patients across the spectrum of CKD and ESKD have demonstrated that low BMD at the hip and forearm do predict incident fractures.^80^, ^81^, ^82^, ^83^ These studies reported that the WHO *T*‐scores perform similarly in patients with and without CKD, with regard to fracture prediction, and resulted in the revised KDIGO recommendation to include BMD measurement in patients with CKD 3‐5D to assess fracture risk.

In the general population, estimates of fracture prediction are improved by the addition of clinical risk factors to BMD measurements. The Fracture Risk Assessment (FRAX) was developed to provide 10‐year absolute risk of major osteoporotic or hip fracture by combining 10 clinical risk factors, with or without femoral neck BMD, into a fracture risk algorithm.^51^ The clinical relevance of FRAX in CKD remains unclear. In a study from the Canadian Multicentre Osteoporosis Study, 320 individuals with an eGFR <60 mL/min, and 1787 with an eGFR ≥60 mL/min were followed for a mean of 4.8 years.^81^ This study showed that FRAX did not differ in its ability to predict major osteoporotic fractures, despite differences in underlying renal function. It is important to note that the incidence of fractures in the CKD patients was low and most patients did not have evidence of CKD‐MBD. In a cross‐sectional study of 353 patients with CKD (mean eGFR of 28 mL/min), approximately 30% had prevalent fractures; FRAX with femoral neck BMD discriminated those with and without fractures but was not superior to femoral neck BMD alone.^84^ All three study groups (FRAX alone, FRAX with BMD, BMD alone) were better discriminants of fracture than age alone. In a study of 485 Japanese hemodialysis patients, FRAX did not predict increased fracture risk over a 3.3‐year median follow‐up.^80^ It is important to note the relatively short follow‐up of this study, along with concerns about the accuracy of the fracture risk assessment data. In a recent study of 718 hemodialysis patients who were followed for a period of 2 years, the area under the curve (AUC) for FRAX was 0.76 (95% confidence interval [CI] 0.69–0.82) for major fractures and 0.70 (95% CI 0.69–0.84) for hip fractures, and FRAX discriminated fractures better than individual elements in the FRAX algorithm, although this did not include BMD.^85^ These studies suggest that more research is needed to determine the usefulness of FRAX in CKD and ESKD. More specifically, some of the clinical factors included in current FRAX algorithms may not be relevant in these patients and CKD‐specific fracture assessment tools need to be developed. These should incorporate predictors of fracture specific to kidney disease, for example, bone alkaline phosphatase (BSAP) or PTH.^86^

## Assessment of Bone Microarchitecture—High‐Resolution Imaging

An important limitation of DXA is that it does not assess the 3‐dimensional structure of bone. Therefore, high‐resolution imaging methods such as QCT, HR‐pQCT, and micro‐MRI have been developed to provide 3‐dimensional imaging of bone density and microarchitectural aspects of bone quality, including cortical and trabecular volumetric BMD, geometry, microarchitecture, and strength. The TBS, although not a high‐resolution imaging modality, has also emerged as an important tool in the assessment of bone microarchitecture and will be discussed below. Micro‐MRI assessment of bone microarchitecture has been evaluated in general and kidney disease cohorts; however, studies of fracture discrimination are lacking, as such no further discussion has been included in this review.^87^, ^88^, ^89^, ^90^, ^91^, ^92^, ^93^

**Figure 2:**
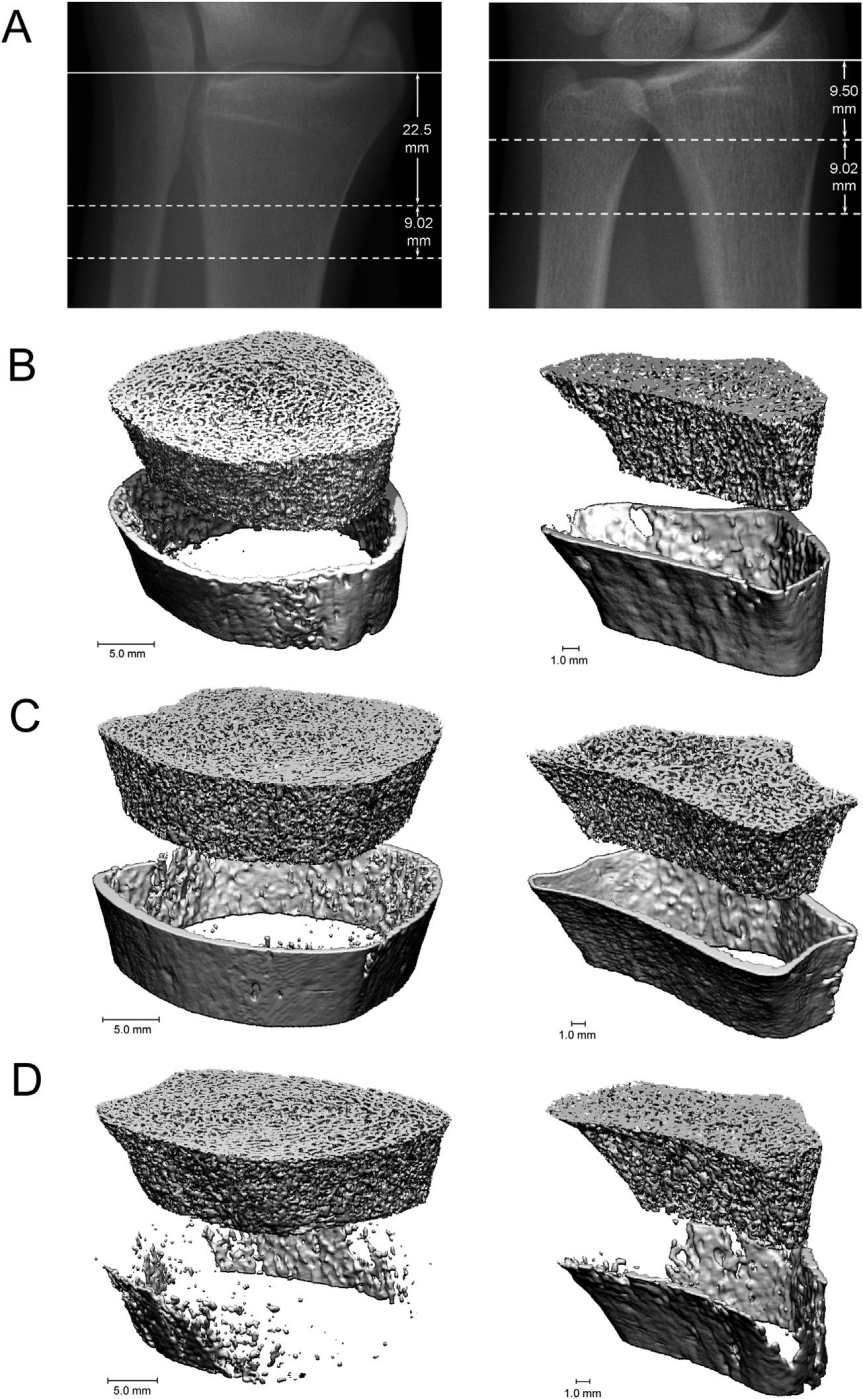
HR‐pQCT provides detailed images of bone microarchitecture at the radius (left) and tibia (right). Scout view (*A*) reference line position (solid line) and the measurement site (dotted line). Images from healthy, postmenopausal white female (*B*). Images from female with CKD, no fractures (*C*). Images from female with CKD and prevalent fractures (*D*). Reprinted with permission from Nickolas et al.^103^

Conventional QCT has a resolution of 300 μm^3^ and quantifies volumetric BMD and cortical and trabecular geometry. In CKD and ESKD, studies utilizing QCT have shown that cortical deficits predominate and these could discriminate and predict future fractures.^94^, ^95^, ^96^ HR‐pQCT has a higher nominal resolution (60 to 82 μm^3^), which allows for quantification of trabecular number, thickness, and separation (Fig. 2). Finite element analysis (FEA) has been used in biomechanics to determine the mechanical behavior (and therefore strength) of bone.^97^ The advent of high‐resolution imaging has allowed FEA to be used in the assessment of bone strength, stiffness, and failure load, either in its entirety or in individual (cortical or trabecular) compartments.^98^, ^99^ Recent developments in HR‐pQCT processing methods have been developed to characterize cortical porosity and trabecular rod and plate structure, which have been strongly associated with bone strength.^100^, ^101^, ^102^

The ability of HR‐pQCT analysis of bone microarchitecture at the distal radius and tibia to assess fracture risk and bone quality has been evaluated in kidney disease. Specifically, HR‐pQCT was reported to discriminate and predict fractures, define abnormalities in bone quality that adversely affect bone strength, and identify microstructural abnormalities that account for reduced bone density as measured by DXA.^103^, ^104^, ^105^, ^106^, ^107^, ^108^ Measures from DXA and HR‐pQCT were associated with prevalent fractures in patients with CKD.^103^, ^104^ Patients with fractures had lower BMD by DXA and lower cortical and trabecular volumetric BMD and thinner cortices and trabeculae by HR‐pQCT. These abnormalities were more severe with longer duration and severity of CKD. A study of 211 men and women with CKD 3–5 assessed the ability of areal BMD by DXA and volumetric BMD by HR‐pQCT to discriminate fractures.^105^ Both tests discriminated fracture status and the addition of HR‐pQCT measures to BMD by DXA did not improve discrimination. In a study of 74 prevalent hemodialysis patients, those with fractures had reduced cortical and trabecular microarchitecture compared with those without fractures.^106^ Changes to bone microarchitecture and calculated bone strength were assessed in 33 ESKD patients and 33 age‐matched controls.^107^ Cortical and trabecular bone microarchitecture and calculated bone strength were altered in ESKD patients; these changes were more pronounced in females. HR‐pQCT has also been used to determine the microarchitectural mechanisms of bone loss in CKD. In a longitudinal study of 54 patients with CKD 2‐5D, the mean annualized loss of bone density by DXA at the radius was 2.9%.^108^ With HR‐pQCT, this was characterized by loss of cortical area, density, and thickness and a significant increase in cortical porosity.

The use of HR‐pQCT in the clinic is not currently practical because of cost and limited availability. Thus, methods to assess bone microarchitecture that can be implanted in the clinic have high potential to assist with the diagnosis and management of patients with metabolic bone diseases. TBS was developed to assess trabecular microarchitecture (ie, bone quality) by using software analysis that measures grayscale homogeneity from lumbar DXA images.^109^ In early studies, TBS has been shown to correlate with trabecular microarchitecture as measured by micro‐computed tomography, HR‐pQCT, and iliac crest bone biopsy.^110^, ^111^, ^112^ TBS was also shown to predict fractures independently of clinical risk factors and areal BMD by DXA^113^, ^114^, ^115^ and has been incorporated into international fracture risk guidelines and predictive algorithms such as FRAX.^116^ TBS has also been investigated in patients with kidney disease. In a recent study of 50 patients with CKD 3‐5D, TBS was associated with trabecular bone volume, width, and spacing, as well as cortical width as measured by bone biopsy.^117^ Luckman and colleagues^112^ reported that TBS measures correlated with both cortical and trabecular microarchitecture by HR‐pQCT in ESKD patients before kidney transplantation and that changes in TBS measurements reflected changes in trabecular microarchitecture and failure load but not cortical microarchitecture 12 months after transplantation. In a Canadian cohort of 1476 patients, those with an eGFR <60 mL/min and a TBS value below the median (<1.277) had a higher 5‐year fracture probability that was independent of BMD and clinical risk factors for fracture (hazard ratio [HR] = 1.62 per SD decrease in TBS).^118^ Further verification and qualification of TBS as a fracture prediction tool in patients with kidney disease is needed.

## Bone Turnover Markers

In patients without CKD, BTMs can be used to assess fracture risk and monitor osteoporosis therapy. In patients with CKD and ESKD, they provide mild to moderate accuracy in the noninvasive assessment of bone turnover and mineralization. In some cases, BTMs can be used instead of bone biopsy to inform antifracture strategies, although the utility of BTMs to improve patient‐related outcomes such as fractures remains unproven. Markers of bone formation (osteoblast function) include BSAP, osteocalcin, and P1NP. Bone resorption markers (osteoclast function) include TRAP‐5b and CTX.

In kidney disease, PTH and BSAP are the most widely tested BTMs to assess bone turnover, and BSAP, vitamin D, calcium, and phosphorus are used to assess mineralization.^8^, ^9^, ^10^, ^67^, ^119^ Generally, extremes of PTH and BSAP identify bone turnover type based on bone biopsy finding in CKD and ESKD. In a study of 132 patients with CKD 3–5, plasma PTH measurements effectively distinguished patients with and without low bone turnover, AUC 0.96 for CKD stages 3 and 4, and 0.86 for CKD stage 5.^119^ In a study of 141 hemodialysis patients, an intact PTH < 420 pg/mL increased the positive predictive value (PPV) for low bone turnover from 74% to 90% in white patients, whereas an intact PTH < 340 pg/mL increased the PPV for low bone turnover from 48% to 90% in black patients.^120^ In 492 ESKD patients, the AUC for discriminating low versus non‐low bone turnover was 0.701 for PTH, 0.757 for BSAP, and 0.718 for PTH and BSAP in combination.^8^ In another study of patients with ESKD, PTH values within the middle range (150 to 300 pg/mL) less reliably identified underlying histology.^121^ In age‐related osteoporosis, BSAP reflects bone formation and correlates with bone histology and other BTMs.^122^, ^123^, ^124^ In a study of 42 ESKD patients, BSAP and PTH were compared with bone biopsy.^122^ BSAP levels were higher in patients with high compared with low turnover (66.9 versus 10.8 ng/mL, *p* < 0.0005), were more strongly correlated with bone formation than PTH, and levels > 20 ng/mL reliably excluded adynamic bone disease. Total ALP has sometimes been used as a surrogate marker of BSAP, particularly in the absence of liver disease. However, ALP has high biological variation (>20%), and a high BSAP can still result in a normal ALP level.^125^, ^126^

PTH and BTMs may also have clinical utility in predicting bone loss and fractures. In a cross‐sectional study of 82 CKD patients, higher levels of PTH and BTMs were associated with lower cortical and trabecular density and increased cortical and trabecular thinning.^104^ In the same study, higher levels of PINP, osteocalcin, and TRAP discriminated fracture. In a prospective 2‐year study of 89 hemodialysis patients, baseline BSAP was strongly associated with loss of BMD cortical mass and volume.^127^ In prospective studies of patients with CKD before (*n* = 52) and after renal transplantation (*n* = 47), the microarchitectural and biochemical mechanisms of bone loss were examined by BTMs, DXA, and HR‐pQCT.^108^, ^128^ Higher levels of PTH, BSAP, osteocalcin, PINP, TRAP, and CTX predicted the loss of cortical area, density, and thickness, increase in cortical porosity, and decreased bone strength. The ability of PTH and BTMs to predict fractures was assessed in a prospective study of 485 ESKD patients. Incident fracture was associated with PTH levels either <150 pg/mL or >300 pg/mL compared with 150 to 300 pg/mL (*p* < 0.01).^80^ In the same study, BSAP was a very useful surrogate marker for any type of incident fracture risk (AUC = 0.766, *p* < 0.0001).

These data suggest that PTH and BTMs may have greater clinical utility in assessing bone quality and fracture risk in CKD and ESKD, in particular when used in conjunction with bone imaging methods. For example, fracture risk can be estimated by DXA. However, deciding on a treatment (vitamin D, calcimimetic, antiresorptive, anabolic) will also require consideration of bone turnover. PTH and BTMs can potentially be used to inform which pharmacologic agent is most appropriate. However, before BTMs can be widely used to manage renal bone disease, significant barriers need to be overcome. BSAP lacks a readily available automated assay, and there are concerns of cross‐reactivity with the liver iso‐enzyme,^129^ there are no validated reference ranges of BTMs in patients with CKD and ESKD, many BTMs are cleared by the kidney (monomeric P1NP, osteocalcin, and CTX) and BTMs have high intra‐ and interassay variability and biological variability.^130^

## Treatment of ROD and Preventing Fractures in CKD

Management of kidney‐associated bone disease for fracture risk reduction is controversial. First, until the release of the 2017 KDIGO guidelines, fracture risk classification in CKD3‐5D was not recommended. Second, there are no antifracture treatments that have been developed specifically for patients with CKD‐MBD. However, since emerging data and anecdotal experience with existing antifracture agents suggest that they are safe, we expect that these agents will be more widely used in patients with CKD 3‐5D, especially as more patients undergo DXA screening. In this section, we will briefly review the antifracture treatments that are in current clinical use and their potential application to patients with kidney disease.

## Management of CKD‐MBD

**Figure 3:**
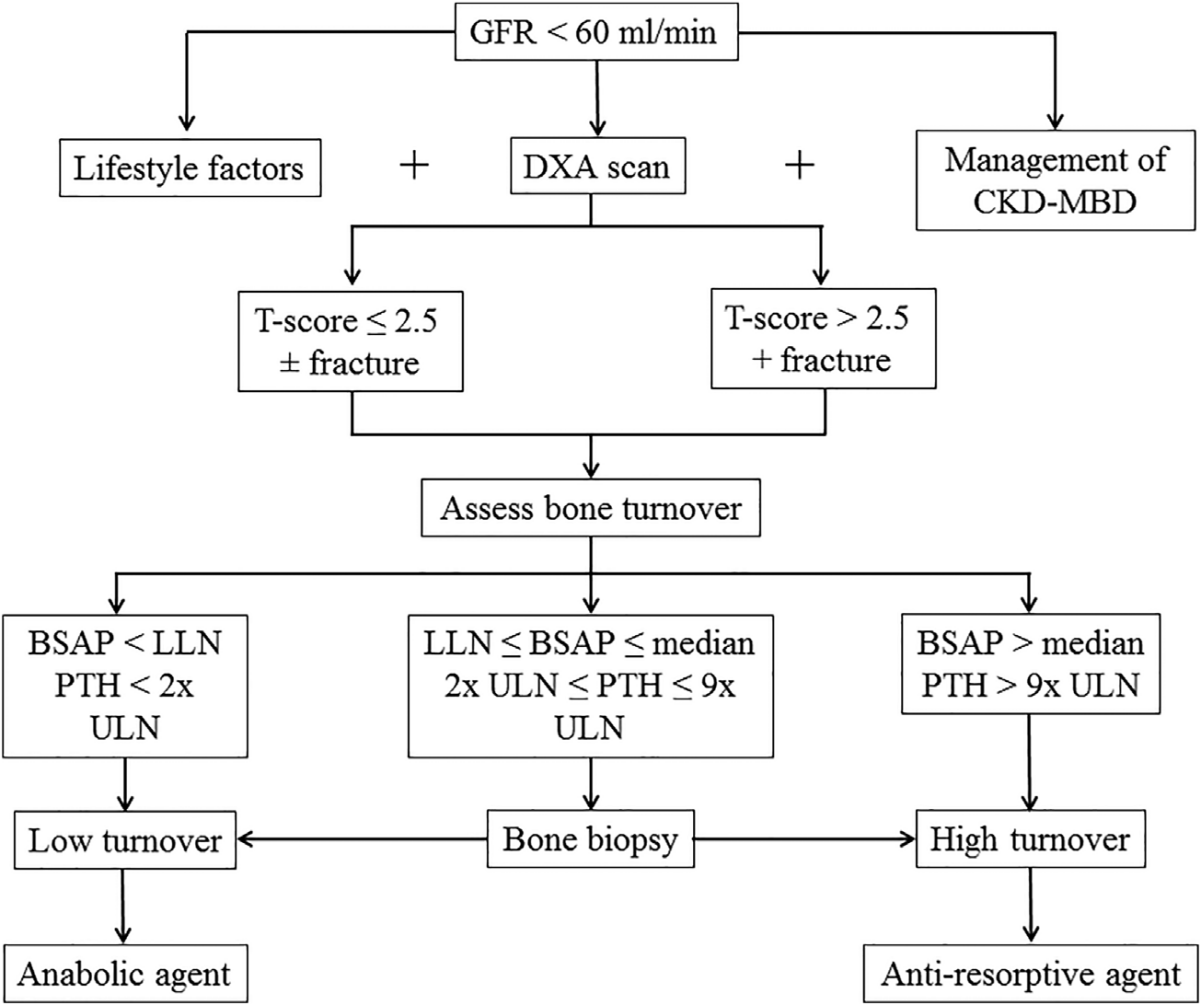
Algorithm for fracture risk screening and initiation of antifracture strategies in patients with CKD. DXA = dual‐energy X‐ray absorptiometry; CKD‐MBD = chronic kidney disease‐mineral and bone disorder; PTH = parathyroid hormone; BSAP = bone‐specific alkaline phosphatase; LLN = lower limit of the normal reference range; ULN = upper limit of the normal reference range. Lifestyle factors include weight‐bearing exercise, cessation of smoking, adequate nutrition, moderate alcohol intake, and fall prevention strategies. Management of CKD‐MBD includes phosphate lowering, vitamin D supplementation (nutritional and active), calcimimetics, and parathyroidectomy. Anabolic agents include teriparatide and abaloparatide. Antiresorptive agents include bisphosphonate and denosumab.

Management of the abnormalities associated with CKD‐MBD must occur before initiating specific antifracture therapy in patients with CKD 3‐5D. A detailed discussion of this is comprehensively articulated in the updated KIDGO guidelines and associated publications.^10^, ^53^, ^131^ In brief, supplementing with vitamin D (nutritional and/or active), lowering phosphate, initiating calcimimetics, and deciding on the need for parathyroidectomy are critically important to treating kidney‐bone disease and can have some antifracture benefits.^132^, ^133^, ^134^, ^135^, ^136^, ^137^ In our opinion, after optimized management of CKD‐MBD, one should consider additive treatment with an agent demonstrated to have antifracture efficacy in the general population (Fig. 3). The updated 2017 KDIGO CKD‐MBD Guidelines clearly endorse the use specific antifracture therapies in CKD 1–2 and to a lesser extent in CKD stage 3 in the absence of abnormalities of CKD‐MBD (recommendation 4.3.1 and 4.3.2).^10^ However, In patients with CKD 3–5 and evidence of CKD‐MBD, the use of osteoporosis therapies is not directly addressed (recommendation 4.3.3): “In patients with CKD G3a–G5D with biochemical abnormalities of CKD‐MBD and low BMD and/or fragility fractures, we suggest that treatment choices take into account the magnitude and reversibility of the biochemical abnormalities and the progression of CKD, with consideration of a bone biopsy (2D).” It is noteworthy that the KDIGO update no longer mandates that a bone biopsy should be obtained before starting osteoporosis treatment, in part because of the increasing experience with the use of osteoporosis medications in CKD patients.

## Antiresorptive Agents

Bisphosphonates and denosumab are commonly used in the treatment of senile and glucocorticoid‐induced osteoporosis; these agents lower remodeling rates and may be helpful in preventing bone loss and fracture in normal and high‐turnover bone disease. These should be avoided in patients with adynamic bone disease, but to date there are no studies in CKD that definitively demonstrate that bisphosphonates cause adynamic bone disease. There are no primary safety and efficacy data on the use of antiresorptive therapies in patients with CKD‐MBD, but there are post hoc analyses of the registration trials in patients with mild to moderate CKD (without biochemical manifestations of CKD‐MBD). These agents can therefore be used in CKD patients who have similar characteristics to the inclusion criteria of the registration trials, but in CKD 3‐5D patients with evidence of CKD‐MBD, further evaluation of the underlying ROD type needs to be undertaken before the use of these agents for fracture prevention.

### Bisphosphonates

Bisphosphonates are selectively taken by osteoclasts, where they inhibit farnesyl pyrophosphate synthase and the synthesis of isoprenoid compounds, which are essential for osteoclast activity, thereby reducing osteoclast‐mediated bone resorption. Oral bisphosphonates have poor bioavailability (<1%); around 50% of the drug is not taken up by osteoclasts and cleared by the kidney. The actual amount of bisphosphonate retained in bone depends on the remodeling space, the GFR, and bone turnover rate.^52^ Given the lack of clinical trial data, oral bisphosphonates are not recommended in patients with eGFR < 30 mL/min because of concerns about excessive accumulation in bone and long‐term suppression of bone remodeling.

However, data from recent fracture intervention trials suggest that these drugs can be safely used in patients with reduced eGFR. In a post hoc analysis of 9 randomized, double‐blind trials comparing risedronate to placebo, 8996 females were identified as having kidney impairment, with the majority having a creatinine clearance of 30 to 80 mL/min.^138^ Risedronate effectively preserved BMD and reduced the incidence of vertebral fractures, and overall and renal function‐related adverse effects were similar in both groups and independent of renal function. Bone biopsy data of 57 patients with renal impairment showed no evidence of adynamic bone disease or mineralization defects. In a secondary analysis of the Fracture Intervention Trial, 581 women (9.9%) had a GFR < 45 mL/min.^139^ Women with a reduced eGFR had a 5.6% increase in total hip BMD, and alendronate increased BMD regardless of eGFR. Treatment with alendronate reduced clinical and spine fractures to a similar degree in those with and without renal impairment. There were no differences in adverse outcomes by renal function. A recent systematic review by Wilson and colleagues^140^ compared bisphosphonate to placebo in six studies (*n* = 1013) of patients with CKD (four of these were in renal transplant recipients).^139^, ^141^, ^142^, ^143^, ^144^, ^145^ Given the heterogeneity of the studies included, the use of bisphosphonates was associated with a nonsignificant reduction in fracture risk and moderate evidence that bisphosphonates may reduce loss of lumbar spine BMD but not femoral neck BMD. A recent post hoc analysis of three Japanese RCTs^146^, ^147^, ^148^ compared risedronate to placebo in 852 patients with an eGFR from 30 to >90 mL/min (with 228 having an eGFR <60).^149^ Patients were analyzed according to eGFR level ≥90, 60 to <90, and ≥30 to <60 mL/min/1.73 m^2^, and lumbar BMD, BTMs (CTX, P1NP, and BSAP) were evaluated at 48 weeks. The increase in lumbar BMD (*p* < 0.001) and inhibition of BTMs (*p* < 0.001) with the use of risedronate did not differ in the eGFR subgroups. There are no data on the optimal duration of bisphosphonate therapy in CKD or ESKD. General population data suggest 3 to 5 years of therapy, with a lack of evidence on fracture reduction beyond 5 years and concerns about atypical femoral fractures.^150^, ^151^, ^152^

### Denosumab

Denosumab is a humanized monoclonal antibody against the Receptor activator of nuclear factor kappa‐Β ligand (RANKL), reducing bone turnover by inhibiting osteoclast proliferation and development. The Fracture Reduction Evaluation of Denosumab in Osteoporosis Every 6 Months (FREEDOM) Trial recruited 7808 postmenopausal women and demonstrated that denosumab administered twice yearly for 36 months was associated with a reduction in the risk of vertebral (68%), nonvertebral (20%), and hip fractures (40%) compared with placebo.^153^ Denosumab is not cleared by the kidney, so unlike bisphosphonates, there is no risk of excess drug accumulation. Its effectiveness and safety in CKD (without evidence of CKD‐MBD) were assessed in a secondary analysis of patients from the FREEDOM trial, where treatment effect was compared across CKD categories (CKD 4 = 73, CKD 3 = 2817, CKD 2 = 4069).^154^ There was no difference in treatment efficacy and adverse effects by renal function, and denosumab increased BMD at the spine and hip and showed a 62% reduction in incident vertebral fractures.

The secondary analysis of the FREEDOM trial did not describe an association between hypocalcemia and decreased renal function. However, case reports and anecdotal experience suggest that this may occur in patients with CKD, hyperparathyroidism, and low vitamin D. In a case series of 8 patients with ESKD and 6 patients with CKD treated with denosumab, 8 patients developed severe hypocalcemia.^155^ In two studies of single‐dose denosumab in CKD and ESKD, hypocalcemia was the most common side effect; however, appropriate pretreatment with calcium and calcitriol was protective of clinically significant hypocalcemia.^156^, ^157^ These and other clinical data suggest that denosumab may have a role in patients with CKD 3‐5D, with appropriate calcium, vitamin D supplementation, and monitoring for hypocalcemia. Denosumab is metabolized in the reticuloendothelial system, and its clinical effect wanes after 6 months; frequency and duration of treatment in CKD 3‐5D remain unclear.

The increasing off‐label use of antiresorptive therapies in kidney disease patients reflects not only the decreasing concern regarding their short‐term safety but also the lack of specific antifracture treatments for patients with CKD‐MBD. No doubt further observational data will continue to guide and refine clinical practice, but ultimately prospective trials are needed to better define safety and antifracture efficacy of these drugs in patients with CKD‐MBD and an eGFR of <30 mL/min/1.73m^2^.

## Osteoanabolic Agents

Osteoanabolic agents are forms of recombinant PTH, and their use in CKD remains controversial. In CKD, high‐baseline PTH levels promote cortical bone loss; therefore, these agents should not be used in patients with high‐turnover bone disease. However, they may increase bone turnover and bone density in patients with adynamic bone disease.

Teriparatide is a recombinant peptide of the first 34 amino acids of human PTH. In health, its antifracture efficacy is caused by an increase in osteoblast number, leading to increased bone formation and thickening of trabecular and cortical bone.^158^ Its efficacy in primary osteoporosis was demonstrated in two large clinical trials.^159^, ^160^ A post hoc analysis of Fracture Prevention Trial examined the safety and efficacy of teriparatide in 736 women with CKD (majority GFR 50 to 79 mL/min, and none <30 mL/min).^161^ Compared with placebo, teriparatide significantly increased lumbar spine and femoral neck BMD and had similar efficacy in preventing vertebral and nonvertebral fractures across all stages of CKD. The main adverse effects were hypercalcemia and hyperuricemia and these were more common with reduced eGFR. In a separate post hoc analysis from the same trial, improvements in BMD were correlated with improvements in trabecular microarchitecture.^162^ Teriparatide has also been used in patients with adynamic bone disease, where it improved BMD at the lumbar spine but not the femoral neck.^163^ A prospective, 48‐week study examined the effect of once‐weekly teriparatide in 22 hemodialysis patients with low BMD and hypoparathyroidism.^164^ Over the treatment period, there was an increase in lumbar spine but not femoral neck BMD. The observed increase in BSAP strongly correlated with improvements in BMD. There was no clinically significant hypercalcemia, and hypotension was the most common adverse event.

Abaloparatide is a newer parathyroid hormone‐related protein (PTHrP) analog, with stronger affinity for the transient state of the PTH1/PTH receptor and a more anabolic profile (compared with teriparatide) given less bone resorption and hypercalcemia.^165^, ^166^ In a study of 222 postmenopausal women, daily abaloparatide (20, 40, or 60 μg) was compared with teriparatide 20 μg or placebo.^167^ BMD was increased at the lumbar spine, femoral neck in a dose‐dependent manner, and to a greater extent than with teriparatide. Abaloparatide was compared with placebo and open‐label teriparatide in a study of 2461 postmenopausal women.^168^ New vertebral fractures occurred less frequently in the active treatment groups, and BMD increases were greater with abaloparatide than placebo. The incidence of hypercalcemia was lower with abaloparatide compared with teriparatide. In a secondary analysis of changes on bone histomorphometry, there was no evidence of abnormal mineralization, bone marrow abnormalities, or presence of excess osteoid.^169^ Patients treated with abaloparatide had lower eroded surface on histomorphometry versus placebo but similar increase in cortical porosity compared with teriparatide. This is supported by a smaller increase in CTX (a resorption marker) with abaloparatide compared with teriparatide. Based on the available data, abaloparatide has the ability to increase bone mass and formation, with less bone resorption and hypercalcemia. It has the potential to become an ideal agent for the treatment of patients with CKD and low to normal bone turnover; however, that data is currently lacking.

Sclerostin is a protein encoded by the SOST gene and secreted by osteocytes. Loss‐of‐function SOST mutations result in a high bone mass phenotype through increased bone formation, and sclerostin has predominantly anti‐anabolic effects on bone through inhibition of the Wnt‐signaling pathway. Inhibition of sclerostin is therefore of great interest in patients with CKD 3‐5D, particularly those with low bone mass and low bone turnover where antiresorptive therapies are contraindicated. In trials of postmenopausal women with osteoporosis, romosozumab increased BMD to a greater extent than existing anabolic agents and decreased vertebral and nonvertebral fractures.^170^, ^171^, ^172^ In another study, comparing romosozumab to teriparatide, there was a greater increase in cortical BMD (compared with trabecular BMD) in the romosozumab arm compared with a reduction in cortical BMD in the teriparatide group.^173^ Increased cardiovascular events were reported in one (but no other) romosozumab study.^171^ As such, it remains unclear whether romosozumab increases cardiac risk, but given the large CVD burden in patients with kidney disease, further study of romosozumab in CKD and ESKD should be suspended until these issues are clarified in future studies. Inhibition of DKK1 is another target for potential novel anabolic agents, although the clinical development of these drugs lags behind that of sclerostin inhibitors. In animal models, inhibition of DKK1 by monoclonal antibodies (DKK1‐ab) generally increased bone formation and mass. In a mouse model, changes of CKD‐MBD including ROD were ameliorated after the administration of DKK1‐ab (in combination with phosphate binder therapy).^174^ In patients with multiple myeloma, administration of DKK1‐ab reduced bone resorption and reduced bone formation.^34^ Finally, the dual inhibition of sclerostin and DKK‐1 leads to synergistic bone formation in rodents and non‐human primates.^175^ These studies have important implications for patients with kidney disease, and clinical studies of DKK1‐ab are needed in CKD and ESKD cohorts.

## Take‐Home Messages and Rethinking Bone Disease in CKD

In CKD patients, fracture rates are more than 10‐fold higher compared with age‐ and sex‐matched individuals without CKD. Although fracture incidence in the general population has fallen over the last two decades, it has increased in patients with ESKD. In the clinic, treatment of CKD‐MBD is focused on the correction of abnormalities associated with parathyroid hormone and phosphate, a strategy that has not mitigated the effects of CKD on fracture risk. Despite advances that have improved our ability to assess fracture risk in the clinic, we continue to lack noninvasive tools that predict turnover and diagnose ROD type, which would inform fracture prevention strategies. Furthermore, despite an ever‐increasing choice of antifracture treatments in the general population, we lack data on their safety and efficacy in CKD 3‐5D. Therefore, it is time to rethink bone disease in patients with kidney disease.

Kidney‐related bone disease is complex and multifaceted, as such any gains are likely to be incremental rather than revolutionary. A starting point could be implementation of the successful fracture screening, prevention, and treatment programs used in the general population to CKD 3‐5D. The 2017 KDIGO guidelines justify the use of DXA, a readily available and inexpensive fracture risk screening tool. Lifestyle measures such as smoking cessation, weight‐bearing exercise, alcohol moderation, and improved nutrition all have proven antifracture efficacy in the general population and could be argued as being even more important in CKD. Vitamin D deficiency is common in CKD and should be routinely supplemented given its skeletal benefits and low risk of adverse consequences. The role of calcium supplementation is less well defined in CKD and ESKD and cannot be recommended in these patients.

Our ability to initiate specific, mostly antiresorptive osteoporosis treatments in CKD 3‐5D remains limited by our inability to effectively assess bone turnover and a historical fear of propagating low turnover and atypical femoral fractures. In the clinic setting, the interpretation and treatment of the current markers of CKD‐MBD should not occur in isolation but in view of the broader endocrine and skeletal disturbances. Although imperfect, PTH and BSAP improve our ability to discriminate bone turnover and some of the microstructural derangements. These should be used with DXA and be further studied with novel algorithms such as TBS that can inform on bone microarchitecture, with only minimal modifications to existing imaging infrastructure. Finally, fracture prediction algorithms such as FRAX should be broadly utilized to increase awareness of fractures; however, these need further validation and refinement to improve their accuracy in patients with kidney disease. Many, if not all, of these measures are feasible and could be readily implemented in most developed countries.

Ongoing work in improving diagnosis of ROD type is needed. Bone biopsy will remain the gold standard; however, given the required expertise, its role will always be confined to specialized tertiary institutions. High‐resolution imaging techniques provide a glimpse of the future, where imaging could obviate the need for a physical biopsy. However, today, even the most advanced high‐resolution imaging techniques remain inadequate at evaluating bone turnover and mineralization, both of which remain essential to informing treatment decisions. We have no doubt that high‐resolution imaging will continue to evolve and there are countless examples in medicine where technology has diminished the need for invasive diagnostic tests but seldom have these been superseded. Therefore, we believe that bone biopsy will remain an important diagnostic and research tool, such as in validating studies of high‐resolution imaging techniques. As such, its availability should be fostered and rationally coordinated across regions and health care networks.

CKD patients are historically disadvantaged by their exclusion from large general population clinical trials, and this is clearly the case in osteoporosis. Much of the current data come from secondary analyses of large osteoporosis trials, where patients were excluded if they had evidence of CKD‐MBD. The safety and efficacy of existing and novel agents in CKD 3‐5D remain unclear, and studies of these drugs are urgently needed in these cohorts. Future studies also need to target patients at various stages in the evolution of renal bone disease; for example, prevention of bone loss in CKD, reduction of fractures in ESKD, as well as any effects on the systemic aspects of CKD‐MBD. For this to occur, stronger advocacy from kidney societies and a paradigm shift from drug companies around the world are required. As physicians, we need to recognize the burden of fractures and bone disease in our patients and work tirelessly to promote better awareness and utilization of available diagnostic and therapeutic resources, such as greater off‐label use of osteoporosis medications in CKD 3‐5D. To this end, collaborations with our endocrinology colleagues should be fostered in both the research and clinical settings. In the words of Lao‐Tzu, “A journey of a thousand miles begins with a single step.” No doubt the road ahead is long, harboring many challenges and false starts; however, to accept the status quo is simply not an option.

## Disclosures

MD has nothing to declare. TN has participated in a Scientific Advisory Board for Amgen. He has received grant support from Amgen for an Investigator Sponsored Study.
